# Oral Probiotic Microcapsule Formulation Ameliorates Non-Alcoholic Fatty Liver Disease in Bio F_1_B Golden Syrian Hamsters

**DOI:** 10.1371/journal.pone.0058394

**Published:** 2013-03-12

**Authors:** Jasmine Bhathena, Christopher Martoni, Arun Kulamarva, Catherine Tomaro-Duchesneau, Meenakshi Malhotra, Arghya Paul, Aleksandra Malgorzata Urbanska, Satya Prakash

**Affiliations:** Biomedical Technology and Cell Therapy Research Laboratory, Department of Biomedical Engineering and Artificial Cells and Organs Research Centre, Faculty of Medicine, McGill University, Montreal, Québec, Canada; Wageningen University, The Netherlands

## Abstract

The beneficial effect of a microencapsulated feruloyl esterase producing *Lactobacillus fermentum* ATCC 11976 formulation for use in non-alcoholic fatty liver disease (NAFLD) was investigated. For which Bio F_1_B Golden Syrian hamsters were fed a methionine deficient/choline devoid diet to induce non-alcoholic fatty liver disease. Results, for the first time, show significant clinical benefits in experimental animals. Examination of lipids show that concentrations of hepatic free cholesterol, esterified cholesterol, triglycerides and phospholipids were significantly lowered in treated animals. In addition, serum total cholesterol, triglycerides, uric acid and insulin resistance were found to decrease in treated animals. Liver histology evaluations showed reduced fat deposits. Western blot analysis shows significant differences in expression levels of key liver enzymes in treated animals. In conclusion, these findings suggest the excellent potential of using an oral probiotic formulation to ameliorate NAFLD.

## Introduction

Non-alcoholic fatty liver disease (NAFLD) is a potentially severe condition that comprises a spectrum of conditions characterized by vesicular fatty change in the liver in the absence of excessive alcohol consumption [1]. NAFLD is associated with increased central adiposity, triglycerides (TG), uric acid, insulin resistance, hyperlipidemia and low levels of high-density lipoprotein (HDL) [2], [3]-. NAFLD prevalence estimates in the general population range from 17–33%, and increases to 100% in the obese population [1]. Other than lifestyle and diet modifications, there is no universally proven treatment [4], [5]. The only available recommendation is avoidance of hepatotoxins and aggressive management of associated co-morbid conditions.

Probiotic bacteria are live microorganisms which confer health benefits when administered in adequate amounts [5]–[14]. These microorganisms need to survive harsh factors encountered in the gastro-intestinal tract and be viable in high concentrations to bestow health benefits on the host. Microencapsulation in specialized ultra-thin semi-permeable polymer membranes has been successfully shown to protect live bacterial cells in oral and other delivery applications [14]–[18].

Ferulic acid [4-hydroxy-3-methoxycinnamic acid] (FA) is considered a potential chemotherapeutic agent as it has the capacity to lower cholesterol and exhibits anti-oxidant associated protective effects [19], [20]. Feruloyl esterase (FAE) causes the hydrolytic release FA from its bound state in most foods (bran cereals, whole grain products, fruits, vegetables, tea, coffee, wine and beer). FAE activity is commonly found in microorganisms in the rumen and in different bacterial genera present in the human and animal gut [21]. FA can be released from its fibre-bound state by the human intestinal microbiota. The presence of FAE activity has been demonstrated in the large intestine of rats and humans and is largely responsible for the release and bioavailability of FA in mammals [22]–[25]. Although there is a partial released of dietary fibre-bound FA by colon micro-organisms, the concentration of the released FA is too low to act as a chemopreventive agent [26], [27]. Because the intestinal FAE is very likely the major route for the release of FA in vivo, the site and levels of this enzyme are critical factors influencing the bioavailability of FA. The presence of FAE activity, able to release antioxidant compounds that can be absorbed in the gut [28], was therefore used as a specific criterion for the selection of the probiotic microorganism.

We have previously studied the activity of known FAE-producing lactic acid bacteria, and selected *Lactobacillus fermentum* ATCC 11976 because of its strong FAE activity [29]. This probiotic was then employed to show that oral ingestion of FAE producing microencapsulated lactic acid bacteria increases availability of FA via supplementation of FAE in the gut [18], [30]. The lipid lowering and antioxidant properties of FA therefore depend on its availability for absorption, the concentration of the released FA and its subsequent interaction with target tissues [19], [20], [26].

In a previous study we have used a computer-controlled simulated human GI model that closely mimics *in vivo* conditions. Microencapsulated FAE-producing bacterial cells were shown to increase their viable counts and the ability to de-esterify feruloyl ester over a 10 hour period under simulated intestinal conditions, after passing through the acidic stomach environment [30]. Another study evaluated the effect of oral administration of the FAE-producing strain *L. fermentum* CRL1446 on the intestinal FAE activity and oxidative status in Swiss albino mice. The determination of basal FAE activity levels in non-treated mice showed that this activity was mainly located in intestinal mucosa, being similar in the small and large intestine [21]. Their results showed that the effect of *L. fermentum* CRL1446 administration on intestinal FAE activity was time and dose dependent. The authors speculated that not only might *L. fermentum* CRL1446 supply exogenous FAE enzymes into the gut, it could stimulate the FAE activity of colonic microbiota via an indirect effect. They further hypothesized that *L. fermentum* CRL1446 could also stimulate the FAE activity of epithelial cells. Nevertheless, FAE induction mechanisms in the gut have not yet been elucidated.

The present research, for the first time, investigates the potential of orally delivered, microencapsulated FAE producing natural *Lactobacillus fermentum* ATCC 11976 bacterial cells in the management of NAFLD in pharmaceutically acceptable formulations.

## Materials and Methods

### Bacterial growth media and chemicals

EFA (ethyl ferulate; ethyl 4-hydroxy-3-methoxycinnamate), PLL [poly(L-lysine)] and CaCl_2_ were purchased from Sigma–Aldrich Canada (Oakville, ON, Canada). Commercially available sodium alginate (A2158), extracted from a brown alga [the giant kelp (Macrocystis pyrifera)] was obtained from Sigma–Aldrich Canada. Its viscosity at 2% (w/v) and at 25°C is ∼250 cP (mPa·s). According to the supplier, the alginate comprises approximately 61% mannuronic acid and 39% guluronic acid and has a molecular mass ranging from 12 to 80 kDa. MRS (De Man–Rogosa–Sharpe) broth was obtained from Difco (Sparks, MD, U.S.A.). The water was purified with an EASYpure Reverse Osmosis System and a NANOpure Diamond Life Science (UV/UF) ultrapure water system from Barnstead/Thermoline (Dubuque, IA, U.S.A.). All other chemicals were purchased from commercial sources and were of analytical or HPLC grade.

### Bacterial strains and culture conditions

*L. fermentum* ATCC 11976 is a known FA-producing bacterium from Cedarlane Laboratories (Burlington, ON, Canada). The bacterium was stored, cultured and microencapsulated in alginate-poly-l-lysine-alginate semi-permeable microcapsules (MWCO 60–70 kDa), as follows. The bacterial culture was stored refrigerated in Difco's MRS medium containing 30% (v/v) glycerol at −80°C and serially propagated three times in fresh MRS medium before experimental use. *L. fermentum* ATCC 11976 was then cultivated in MRS-EFA broth [MRS supplemented with 10% (w/v) EFA in DMF (dimethylformamide)]. A 1% (v/v) inoculum was used, and incubations were performed at 37°C for 20 h under microaerophilic conditions (5% CO_2_).

### Microencapsulation of bacterial cells and their enumeration

Microencapsulation was carried out with 36-h-old cultures of the bacteria grown in MRS-EFA broth by using an Inotech encapsulator (Inotech Biosystems International, Rockville, MD, U.S.A.). The cultures were centrifuged at 7000 g for 20 min at 15°C. The supernatant was decanted off. The pelleted CWW (cell wet weight) was resuspended in PS [physiological saline (0.9% (w/v) NaCl)], pooled and gently added to 60 mL of a lightly stirred sterile 1.8% (v/v) sodium alginate solution. Encapsulation parameters were kept constant: (cell load, 4.17 g CWW/100 ml of alginate; nozzle diameter, 300 µm; vibration frequency, 918 Hz; syringe pump speed, 9.64 ml/min; voltage, 1.5 kV; and current 2 A). The beads were allowed to gel for 5 min in a gently stirred sterile CaCl_2_ solution (0.1 M), followed by a wash with PS. It was consequently coated for 10 min with 0.1% (w/v) sterile PLL followed by a wash with PS. A final coat of 0.1% (w/v) sterile alginate for 10 min followed, trailed by a final wash with PS. Bacteria-free empty capsules were prepared as controls. This procedure was performed in a Microzone Biological Containment Hood (Microzone, Ottawa, ON, Canada) to assure sterility. The microencapsulated lactobacilli were stored in minimal solution (10% (v/v) MRS and 90% (v/v) PS) at 4°C until further use. The bacterial numbers of the Lactobacillus strain in the microcapsules were determined by plate counts on MRS agar. The bacteria were pour-plated at the end of the refrigerated storage time. For each bacterium, storage fluid was aspirated; the microcapsules were crushed and resuspended in 0.9 mL of PS. Samples were serially 10-fold diluted in diluent. Triplicate plates were inoculated with 0.5 mL samples from the appropriate dilutions and incubated under 5% CO_2_ at 37°C for 72 h. The microcapsules contained, on an average, 10^11^ cfu (colony-forming units)/mL of bacteria. Morphological studies by microscopic analysis revealed that the mean capsule diameter was 602±30 µm for both empty and bacteria-loaded capsules and the encapsulated bacterial cells appeared to be evenly distributed within the membrane.

### Animals, diet and NAFLD

Male Bio F_1_B Golden Syrian hamsters, 8 weeks old (BioBreeders, MA, USA) with an average body weight of 90 g were housed in a climate-controlled space with inversed, alternating light and dark cycles (12:12-hr light– dark cycle; lights on at 19:00). All experimental protocols complied with the Animal Care Committee of McGill University and Canadian Council on Animal Care guidelines. The animals were acclimatized for 2 weeks before the start of the experiment. They were fed commercial rodent ration (LabDiet® Rodent Laboratory Chow 5001, Purina Laboratories, St. Louis, MO, USA) and water. Baseline values of serum total cholesterol (TC) were determined at the end of acclimatization from 14-hour food-deprived hamsters. These values were used to randomize animals into treatment groups. NAFLD was induced in hamsters, as described previously [31]. Briefly, the animals were fed a synthetic, purified hyperlipidemic methionine-deficient and choline-devoid diet (Purina Laboratories, St. Louis, MO) for 5 weeks, following a switch to a semi-purified hyperlipidemic methionine-adequate and choline-deficient diet (Purina Laboratories, St. Louis, MO, USA) [31].

### Experimental protocol

To investigate the therapeutic efficacy of the formulation, animals in the treatment group (*n* = 12 per group) were orally gavaged 1 mL of microcapsule formulation (*L. fermentum* ATCC 11976 cells at 11.51 log cfu/mL) twice daily and the control group (*n* = 12) with empty microcapsules, for 12 weeks, using 18G/50 mm with 2.25 mm ball diameter stainless steel gavage needles. Diet consumption and body weight were monitored. Fasting blood samples were collected every 14 days via the saphenous vein. The hamsters were euthanized by CO_2_ asphyxiation and blood was withdrawn by cardiac puncture. The whole liver was excised and flash frozen in liquid nitrogen.

### Metabolite identification using mass spectrometry

25 µL of serum was diluted using 20% (v/v) methanol to a volume of 1 mL. Mass spectra were recorded on a Finnigan LCQ DUO ion trap mass spectrometer (Fisher Scientific, Ontario, Canada) using electrospray (ESI) in the positive mode. The ion-spray voltage was maintained at 4.5 KV, nitrogen was used as the nebulising sheath gas and the heated capillary temperature was kept at 200°C and scanning from mass 50 to 500.

### Histology analysis

Cryopreserved livers were ultra-sectioned (4–5 µm thickness), stained for fat with ORO and examined under light microscopy. Coded histologic slides of livers were scored for fat infiltration using MATLAB (The MathWorks, Inc., MA, USA) as follows: no visible fat: score 0; up to 10% of liver surface infiltrated by fat: score 1; 10% to 30% fat: score 2; 30% to 50% fat: score 3; and >50% fat: score 4. Infiltration was classified as micro-vesicular, macro-vesicular, or mixed.

### Determination of key proteins in liver cholesterol metabolism

Liver tissue was lysed, homogenized and the supernatant was processed for extraction of hepatic lipids and proteins, according to the procedure previously described [32]. The sample was analyzed for levels of 3-hydroxy-3-methylglutaryl coenzyme A (HMG-CoA) reductase, cholesterol 7α-hydroxylase (CYP7A1), acyl-CoA cholesterol acyltransferase-1 and -2 (ACAT-1 and ACAT-2) and carboxyl ester lipase (CEL). Protein concentration from the liver extract and serum was determined by *RC DC* Protein Assay (Bio-Rad Laboratories, CA, USA). Abundance in the liver extract and the serum concentration of lecithin cholesterol acyltransferase (LCAT) was determined by Western blot. HMG-CoA reductase goat polyclonal antibody (1∶100), goat polyclonal anti-CYP7A1(1∶200), goat polyclonal anti-LCAT antibodies (1∶200) from Santa Cruz Biotechnology (Santa Cruz, CA, USA), rabbit polyclonal anti-ACAT-1(1∶500) (Novus Biologicals, Littleton, CO, USA), rabbit polyclonal anti-ACAT-2 (1∶100) (generously provided by Professor Lawrence L. Rudel, Department of Biochemistry and Comparative Medicine, Wake Forest University, Winston-Salem, NC.) and goat polyclonal anti-CEL (1∶200) (Santa Cruz Biotechnology) were used as primary antibodies. The Western blots were quantified using ImageJ 1.40 (Rasband, W.S., ImageJ, U. S. National Institutes of Health, Bethesda, MD, USA, http://rsb.info.nih.gov/ij/, 1997–2008).

### Clinical chemistry analysis

Serum lipids (TC, HDL cholesterol and TG), glucose, uric acid and C-reactive protein (CRP) and liver function tests for albumin, ALT, aspartate transaminase (AST), alkaline phosphatase (ALP) and Gamma glutamyl transpeptidase (GGT) were assayed using a Hitachi 911 clinical chemistry autoanalyzer (Roche Diagnostics, USA) with reagent kits from Roche Diagnostics (Laval, QC, Canada). Serum free cholesterol (FC) and non-esterified fatty acids (NEFA) were estimated using kits (Wako Chemicals USA, Inc. VA).

Serum insulin was measured by an Enzyme Immunoassay from SpiBio (Massy, France). Fasting plasma insulin and glucose were used to calculate insulin resistance from the homeostasis model assessment for insulin resistance (HOMA-IR) [(fasting glucose * fasting insulin)/22.5] [33].

Hepatic TC, FC, TG, NEFA and phospholipid concentrations were determined enzymatically. Hepatic and serum esterified-cholesterol (EC) concentrations were calculated as the difference between TC and FC concentrations. Total hepatic lipid content was calculated as the sum of FC, EC, TG, NEFA and phospholipids. Total neutral lipids were determined as the sum of FC, EC, TG and NEFA.

### Statistical analysis

Statistical Analysis System (SAS Enterprise Guide 4) (SAS Canada, ON) was used for statistical analysis. Data are presented as mean ± standard deviation (s.d.). One- way analysis of variance (ANOVA) or Student's *t* test were used to compare mean differences between the control and the experimental groups, as appropriate. Differences between variables were considered significant at a 95% confidence interval (*P*<0.05).

## Results

### Metabolite identification using mass spectrometry

**Figure 1:**
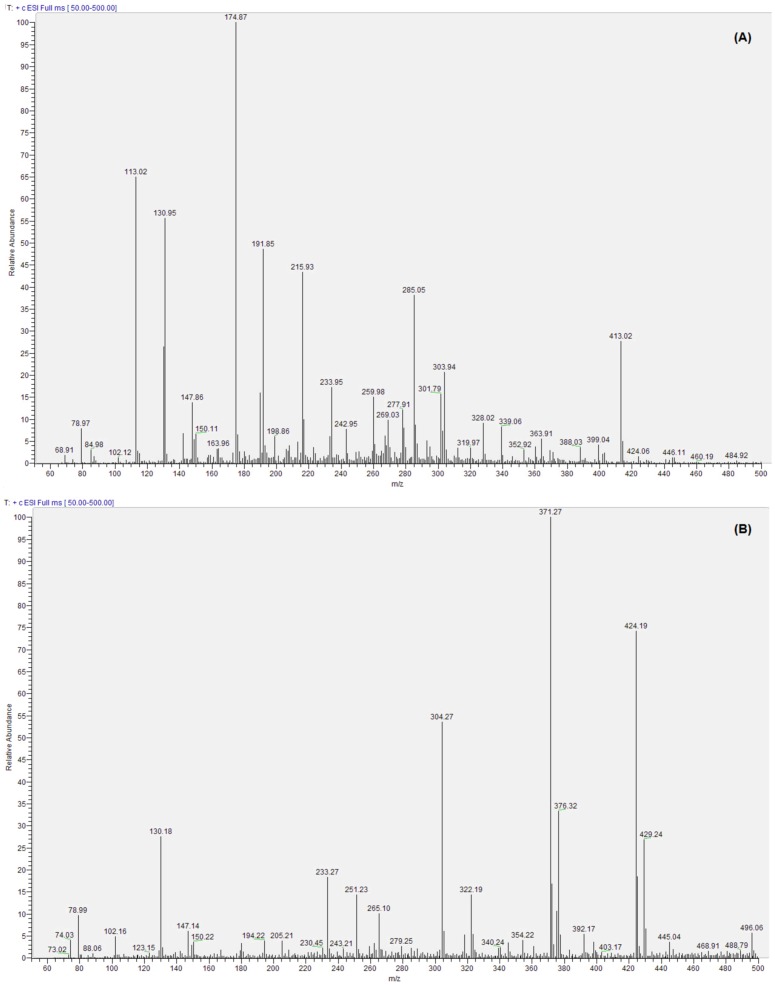
ESI^+^– MS spectrum of hamster serum. (A) control animals (B) animals treated with microencapsulated *L. fermentum* ATCC 11976. The decisive molecular ion [M+1]^+^ detected, only in the treatment group sera, was 371 in the MS spectrum, correlating to the addition of a glucuronic acid (m/z 176) to FA (m/z 194).

Each spectrum was obtained from the end-point serum of the animals. ESI analysis in positive mode was shown as sensitive to the metabolites of interest. Molecular ions of three known metabolites of FA were searched for [34]. The molecular ion [M+1]^+^ detected in the treatment group sera was 371 in the MS spectrum, correlating to the addition of a glucuronic acid (m/z 176) to FA (m/z 194). No free or conjugated forms of FA were detected in control animal sera, as shown in Figure 1.

### Liver histology

**Figure 2:**
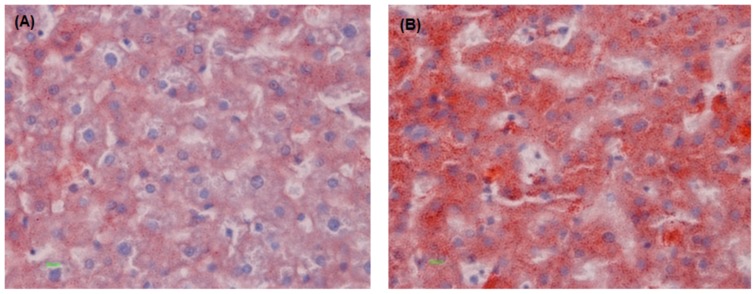
Representative photomicrographs of livers from hamsters after 12 weeks. (A) with microencapsulated *L. fermentum* ATCC 11976 treatment and, (B) without treatment. Microencapsulated *L. fermentum* ATCC 11976-supplemented animals (A) show lesser fat infiltration in the hepatocytes whereas, control animals (B) are filled with 30% greater micro-vesicular fat deposits (Oil Red O, 400X).

Figure 2A and 2B show photomicrographs of liver histology representing the lipid distribution of hamsters administered microencapsulated *L. fermentum* ATCC 11976 for 12 weeks. As expected, fatty liver changes were detected in all animals with hepatocytes comprising micro-vesicles filled with small, fine fat droplets and centrally-placed nuclei. Macro-vesicles, obscuring the nucleus by displacement to the periphery of the cell were present in only two of twelve control hamsters along with concomitant micro-vesicles. The degree of fatty change, evaluated as fatty infiltration scores, was significantly lowered in the treated hamsters (P = 0.0028). The score decreased from 2.4±0.50 in the controls to 1.8±0.50 in the treated group (Table 1).

**Table 1:** Histological fatty-liver scores of hamsters fed hyperlipidemic diet without and with treatment with microencapsulated *L. fermentum* ATCC 11976.

	Hamsters, 12 Weeks on hypercholesterolemic, hyperlipidemic diet	
Score	Control animals (n = 12)	Animals treated with microencapsulated L. fermentum ATCC 11976 (n = 12)
0	0	0
1	0	3
2	7	9
3	5	0
4	0	0
Mean Score ± SD	2.4±0.5	1.8±0.5

### Key metabolic enzymes

**Figure 3:**
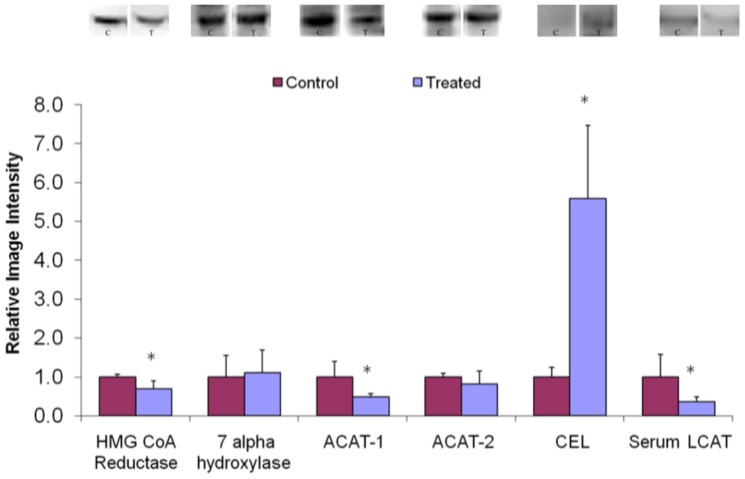
Western blot and group data depicting key hepatic and serum cholesterol metabolism protein abundance in liver of hamsters fed a high-cholesterol high-fat diet, without (control) and with microencapsulated *L. fermentum* ATCC 11976 supplementation. *n* = 12. * *P*<0.05.

Results, in Figure 3, show hepatic tissue HMG-CoA reductase levels in the group treated with microencapsulated *L. fermentum* ATCC 11976, 30.7% lesser than that in control animals (P = 0.027). Furthermore, no significant difference was found in hepatic CYP7A1 protein levels between the treated and control animals. Consequently, the HMG-CoA reductase-to-CYP7A1 ratio, reflecting the balance between cholesterol synthetic and catabolic capacities, was markedly decreased in the treatment group. The treated group showed a significant down-regulation of ACAT-1 (51.1%) compared to the control group (P = 0.043). On the other hand, liver levels of ACAT-2 were virtually identical in both groups. Liver CEL levels were more elevated in the control than the treated hamsters (82.1% down-regulation) (P = 0.014). Data in Figure 3 shows a significant difference (63.4%) in serum LCAT levels between the treated and control hamsters (P = 0.0048).

### Hepatic lipids

**Figure 4:**
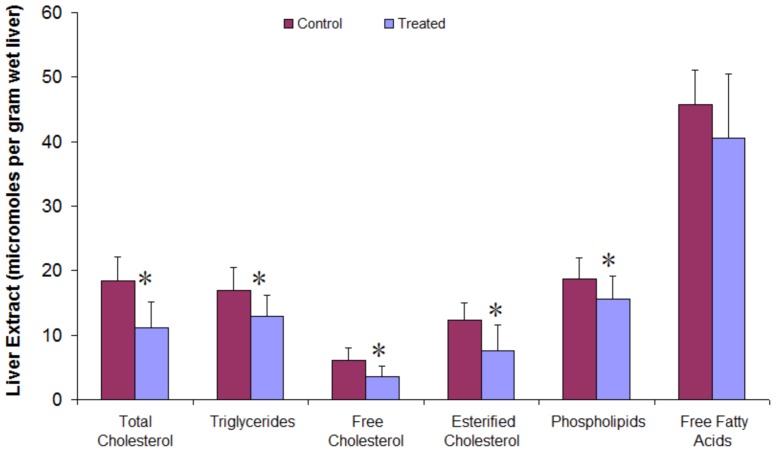
Pre-clinical efficacy of microencapsulated *L. fermentum* ATCC 11976 on hepatic neutral lipids and phospholipids in hamsters. *n* = 12. * *P*<0.05.

Results in figure 4, show a significant lowering effect of the gross liver weight in treated animals (3.91±0.39 g/100 g body weight) compared to control animals (4.63±0.25 g/100 g body weight) (P<0.0001). Animals receiving microencapsulated *L. fermentum* ATCC 11976 had a significant reduction in total liver lipid concentrations (80.10±13.17 µmol/g liver), compared to control animals (99.75±11.41 µmol/g liver) (P = 0.001).

Examination of the liver lipid-extract revealed that about 80% of the total hepatic lipids were neutral lipids, while the rest comprised phospholipids. The treated group had total neutral lipids of 64.48±10.78 µmol/g liver, significantly lower than the control group (80.98±8.55 µmol/g liver) (P = 0.0006). The decrease in neutral liver lipids affected mostly the hepatic cholesterol and triglyceride levels. Free and esterified cholesterol concentrations were 3.52±1.66 and 7.60±3.93 µmol/g liver in treated animals and 6.11±1.90 and 12.25±2.79 µmoles/g liver, respectively in controls (P = 0.0022 and P = 0.0039, respectively). The triglyceride concentration was 12.85±3.27 µmol/g liver tissue in treated animals, compared with 16.87±3.69 µmol/g liver in controls (P = 0.011).

While the probiotic formulation decreased triglyceride and cholesterol concentrations in treated hamsters, the effects on non-esterified fatty acids were less (P = 0.14). The hepatic phospholipid content of the treated hamsters (15.62±3.57 µmol/g liver) was significantly less than the controls (18.77±3.21 µmol/g liver) (P = 0.038). A greater accumulation of hepatic neutral lipids and phospholipids in the control animals was observed compared to treated animals (Figure 4).

### End-point fasting serum metabolites

At the experimental end-point, significant differences were observed in serum TC, HDL, EC, NEFA, uric acid and serum fasting insulin, with a decrease in HOMA-IR in treated animals as compared to the control group (Table 2). Furthermore, serum FC, TG, glucose and CRP levels were not significantly different between the two groups (Table 2).

**Table 2:** Serum metabolite levels in hamsters gavaged empty capsules (control) and microencapsulated *L. fermentum* ATCC 11976 (treatment).

		Hamsters, 12 Weeks on hypercholesterolemic, hyperlipidemic diet			
End-point serum metabolites	Unit	Control animals, Empty capsules (n = 12)	Animals treated with microencapsulated L. fermentum ATCC 11976 (n = 12)		P-value
TC	mmol/L	23.18±8.74	14.33±8.25	0.024	
HDL	mmol/L	2.65±0.16	1.67±0.21	0.0001	
FC	mmol/L	1.55±1.13	1.71±1.08	0.76	
EC	mmol/L	21.94±9.79	12.91±8.69	0.033	
TG	mmol/L	8.36±1.75	6.28±3.37	0.095	
NEFA	mmol/L	3.33±0.89	2.65±0.42	0.029	
Glucose	mmol/L	14.21±5.98	15.41±4.36	0.59	
Insulin	ng/mL	1.40±0.79	0.59±0.35	0.0042	

### Liver Function Tests

The liver function test results are shown in Table 3. Total serum albumin, AST and ALP of hamsters was not affected by treatment with microencapsulated *L. fermentum* ATCC 11976 (P>0.05). On the other hand, ALT and GGT values were significantly lower (P<0.05) for the hamsters treated with the probiotic formulation as compared to the control animals.

**Table 3:** Liver function tests in hamsters gavaged empty capsules (control) and microencapsulated *L. fermentum* ATCC 11976 (treatment).

			Hamsters, 12 Weeks on hypercholesterolemic,hyperlipidemic diet		
Parameter	Reference Range	Unit	Control animals, Empty capsules (n = 12)	Animals treated with microencapsulated, L. fermentum ATCC 11976 (n = 12)	P-value
Albumin	35–55	g/L	40.33±16.61	30.88±5.67	0.079
ALT	7–56	U/L	80.46±53.94	28.40±26.86	0.022
AST	5–35	U/L	35.93±12.42	55.29±21.49	0.130
ALP	20–126	U/L	82.36±8.30	82.28±32.06	0.990
GGT	8–78	U/L	24.05±5.29	12.03±4.83	<0.0001

## Discussion

This study evaluated the efficacy and investigated the potential mode(s) of action of microencapsulated *L. fermentum* ATCC 11976 to reduce cholesterol accumulation in the liver of hamsters with diet-induced NAFLD.

FA, in conjugated form, is continuously introduced into circulation due to metabolic activities of the bacterial formulation. From there, it is distributed to the liver and other tissues to exert beneficial effects. FA is rapidly excreted via the urine but the conjugated FA metabolite is secreted into bile and enters the enterohepatic circulation [35]. This may prolong FA and FA-metabolite bioavailability, explaining the presence of glucuronide metabolite in the serum of fasted, treated animals (Figure 1). The absence of corresponding metabolites in the serum of control hamsters is evidence of active FAE in the GI tract (Figure 1).

Oral feeding of the microencapsulated probiotic effectively reduced fat infiltration in the liver, despite continuous feeding of a high-fat, high-cholesterol diet. Hepatic-fat lowering was attributed to the redistribution of lipid depots in the body rather than intestinal mal-absorption of fat. Although liver weight was significantly reduced in treated hamsters, no histopathological signs of inflammation or tissue damage were observed. The data suggests that a higher level of metabolic impairment of fatty acid oxidation may exist in control hamsters. In the liver itself, lipid (cholesterol, triglyceride and phospholipid) metabolism was altered due to bacterial metabolism. The major decrease in hepatic neutral lipids was observed in the cholesterol fraction, whereas the proportion of TG remained stable and NEFA significantly increased. FC accumulation in the mitochondria, but not TG and NEFA, is known to sensitize hepatocytes to cytokine-induced apoptosis and disease progression [36]. The probiotic formulation is thus thought to reduce cholesterol augmentation in the mitochondria and restore antioxidant levels, offering some protection from liver damage. The increased neutral lipids and NEFA in the control animal livers is consistent with decreased oxidative capacity, creating favorable conditions for the “second hit” which may cause progression to severe NAFLD stages.

The decrease in liver cholesterol of treated hamsters can be related to the changes in key liver proteins. The reduction in liver HMG-CoA reductase may be due to regulation of feedback control of cholesterol synthesis mechanisms that operate during cholesterol supplementation. The comparatively higher expression of hepatic ACAT-1 in the control group may account for the elevated hepatic EC content, as its main function is to prevent the accumulation of free cholesterol within cell membranes by conversion to cytoplasmic lipid droplets [37], [38]. In contrast, levels of ACAT-2, which play a role in hepatic packaging, production, and secretion of very low density lipoproteins (VLDL) [37], were unchanged. Typically, expression of CYP7A1 is augmented by increased free-cholesterol in hepatocytes [39]. In this study, unchanged hepatic CYP7A1 levels and reduced hepatic HMG-CoA reductase were associated with a 42% reduction in the relative ratio of HMG-CoA reductase-to-CYP7A1. CEL is the key enzyme required for releasing free cholesterol from intracellular esterified cholesterol storage when cellular-free cholesterol levels are depressed [37]. LCAT deficiency may have disrupted the reverse-cholesterol transport pathway and, consequently, a reduction in either abundance of serum HDL cholesterol, the particle distribution of HDL or the lipid composition of circulating lipoproteins [40].

The unchanged rate of cholesterol depletion, owing to continued secretion of VLDL particles, bile acids and biliary cholesterol (normal levels of ACAT-2 and CYP7A1), and the relative reduction of cholesterol synthesis and storage due to down-regulation of HMG-CoA reductase and ACAT-1, may have caused a paucity of free cholesterol in the cellular pool of the liver. This suggests that up-regulation of CEL may be a compensatory response to decreased hepatic tissue FC concentrations. Since CEL was up-regulated, the stored cholesteryl esters may have been de-esterified to increase available FC as substrate for CYP7A1, accounting for the decrease in fat content of the treated hamster livers. Export and catabolism of the liver cholesterol was thus shown to exceed import and synthesis in the livers of treated animals.

In NAFLD, when adipose tissue becomes resistant to insulin, serum lipoprotein levels alter, and influx of NEFA to the liver increases, diverting fatty acids into storage rather than secretion [40]. When storage increases, the intra-hepatic NEFA become more amenable to lipid peroxidation whose by-products can impair mitochondrial function and promote hepatic inflammation [36], [40]. In the presented study, the NEFA content of the treated hamster liver did not significantly change, whereas the serum of treated hamsters showed a significant reduction in NEFA. The predominant increased flux of fatty acids in NAFLD may occur between the visceral fat and liver via the portal circulation. Measurements of NEFA in the systemic serum may, therefore, inaccurately reflect levels in the portal circulation presented to the liver. In contrast, the TG content of the treated hamster livers markedly decreased while the serum content did not differ from that of the controls. Thus, the other mechanism for reducing lipid levels in NAFLD may be related to decreasing hepatic fatty acid synthesis and increasing TG export from the liver.

The balance between cholesterol biosynthesis and catabolism is a critical determinant of serum lipid levels. Following treatment, the hamsters showed a considerable decrease in serum TC, although serum FC was unchanged, potentially attributed to a demand by the liver for accessible cholesterol for VLDL formation or conversion to bile salts. Because serum LCAT values were suppressed, it is obvious that there would be a concurrent decrease in serum HDL, however, serum HDL concentrations in treated animals remained well within the normal reference range. As reported before, lowered concentrations of serum HDL translated into diminished serum EC concentrations [40]. Cholesterol metabolism is complex, however, such that blood levels might not always reflect the levels of cholesterol in the liver.

Insulin not only triggers the development of NAFLD but also worsens its course. We show that fasting serum insulin levels are closely associated with NAFLD. Our results are also similar to those of Kelley et al., who found a significant correlation between plasma-NEFA, insulin sensitivity and NAFLD [39]. Thus, insulin resistance represents a target for a therapeutic approach.

CRP levels are known to correlate significantly with NAFLD histological features (degree of steatosis, necro-inflammation and fibrosis); giving support to the earlier hypothesis that NAFLD is associated with low-grade systemic inflammation [41]. However, CRP levels did not differ between the treated and control hamsters, despite changes in liver fatty infiltration.

NAFLD is also characterized by elevated serum uric acid levels, an independent predictor of NAFLD since they increase progressively with the presence and severity of fibrosis [5], [42]. Elevated uric acid serum levels in NAFLD is within the context of insulin resistance since urinary uric acid clearance decreases in proportion to increases in insulin resistance, leading to an increase in serum uric acid concentration [42]. In our study, a decrease in serum uric acid concentrations in the hamsters treated with microencapsulated *L. fermentum* ATCC 11976 reflects the corresponding increase in insulin sensitivity and thereby, improvement in liver histopathology.

ALT is the enzyme known to correlate with liver fat accumulation and is a biomarker of NAFLD, along with GGT [29]. Serum levels of ALT and GGT are positively associated with fasting insulin levels required to maintain normal hepatic glucose production [43]. In our study, the treatment demonstrated a normalization of ALT and GGT values, although showing no particular effect on serum AST levels.

The data from our study suggests the need for well-controlled trials. No randomized clinical trials have so far been identified which support or refute probiotics for patients with NAFLD [44]. Preliminary data from two pilot non-randomized studies suggest that probiotics may be well-tolerated, may improve conventional liver function tests, and may decrease markers of lipid peroxidation. This probiotic formulation has also shown potential as a therapeutic for metabolic syndrome [45]. The positive effects of the microencapsulated *L. fermentum* ATCC 11976 formulation treatment on liver tests, histopathology and on parameters of pathological events related to NAFLD indicate its as a complementary therapeutic approach in fatty-liver disease patients.
